# Imaging of Biliary Involvement in Sarcoidosis: Computed Tomography, Magnetic Resonance Cholangiopancreatography, and Gadolinium Ethoxybenzyl Diethylenetriamine Pentaacetic Acid-Enhanced Magnetic Resonance Imaging Findings

**DOI:** 10.3390/tomography7040065

**Published:** 2021-11-13

**Authors:** Matteo Renzulli, Mario Casavola, Alberto Foà, Carmine Pizzi, Rita Golfieri

**Affiliations:** 1 Department of Radiology, IRCCS Azienda Ospedaliero-Universitaria di Bologna, 40138 Bologna, Italy; mario.casavola@studio.unibo.it (M.C.); rita.golfieri@unibo.it (R.G.); 2 Department of Experimental, Diagnostic and Specialty Medicine-DIMES, University of Bologna, IRCCS Sant’Orsola-Malpighi Hospital, 40138 Bologna, Italy; alberto.foa2@unibo.it (A.F.); carmine.pizzi@unibo.it (C.P.)

**Keywords:** sarcoidosis, biliary tract, cholangiopancreatography, magnetic resonance, multidetector computed tomography, positron emission tomography, liver diseases

## Abstract

Sarcoidosis is a multisystem disease usually affecting the chest, hilar lymph nodes, and lungs, but can potentially involve any organ; therefore, its clinical presentation may vary. Hepatobiliary involvement is rare, and typically asymptomatic; however, it can lead to cirrhosis, and may require liver transplantation. In this report, we present a rare case of a patient affected by sarcoidosis with hepatobiliary involvement. He presented to our hospital complaining of dyspnea triggered by moderate efforts and oppressive thoracic discomfort. Chest X-ray showed multiple bilateral nodular opacities and enlargement of both hilar regions, confirmed by a subsequent total-body computed tomography scan and positron emission tomography, which also revealed cardiac, splenic, and hepatic involvement. Liver function was studied via gadolinium ethoxybenzyl diethylenetriamine pentaacetic acid (Gd-EOB-DTPA)-enhanced magnetic resonance imaging, and magnetic resonance cholangiopancreatography (MRCP) was also performed. The diagnosis of sarcoidosis was finally achieved via liver biopsy, revealing non-necrotizing granulomas in the periportal space. The patient was treated with prednisone per os, with regression of all lesions at all levels. Although other cases of biliary sarcoidosis have been described, this report provides a complete image set of Gd-EOB-DTPA-enhanced magnetic resonance and MRCP images that is lacking in the English literature, and which may be useful for diagnosis.

## 1. Introduction

Sarcoidosis is a multisystem inflammatory disease that generally affects patients aged 40–55 years, without significant gender differences, and with a variable incidence rate based on the country and ethnic group [1]. A recent study from the Mayo Clinic, examining a cohort composed mainly of white people, estimated an incidence rate of 11 per 100,000 people/year [2]. Another study from the United States reported an incidence rate of 8.1 per 100,000 people/year in Caucasians, 17.8 in African Americans, 4.3 in Hispanics, and 3.2 in Asians [3]. In European countries, an incidence of 11.5 per 100,000 people/year has been reported in Sweden [4], and of 4.9 per 100,000 in France [5]. A Korean study reported increase over time in both the sarcoidosis incidence rate and the age of diagnosis—presumably linked to population aging [6]. 

There is a lack of consensus on the etiology of sarcoidosis, and various associations between occupational or environmental exposures and genetic factors have been described. Sarcoidosis is histologically characterized by non-caseating granulomas made up of epithelioid and multinucleated giant cells [7,8,9].

In more than 90% of patients, sarcoidosis affects the lungs and lymph nodes; however, this multisystem disease can affect virtually any organ [10,11,12].

Due to its multisystem diffusion and wide range of clinical presentations, the diagnosis of sarcoidosis is challenging, and is often delayed. In everyday practice, the presumptive diagnosis is reached by correlating clinical and radiological findings, and is then confirmed by histological evidence of non-necrotic granulomas. Computed tomography (CT), 18F-fluorodeoxyglucose positron emission tomography (18F-FDG-PET), and magnetic resonance imaging (MRI) can be used to identify sarcoidosis in various organs, although a gold standard has not been established [13].

Hepatic involvement in sarcoidosis is reported to range from 50% to 80% of cases with systemic disease [14,15].

Nevertheless, in symptomatic patients, the clinical presentation may vary: fatigue is commonly reported [16]; abdominal pain is complained of in 15% [17] of cases, and fever in 3–28% [18]. Jaundice and pruritus are noted in less than 5% [19] of cases, and are usually related to intra- or extrahepatic biliary involvement [17]. Upon radiological examination, enlargement of the liver and spleen can be found in half of patients—especially in the presence of portal hypertension. The latter is reported to cause esophageal varices in up to 78% of cases, rather than severe liver dysfunction, which is an unusual complication. Diffuse hepatic involvement can lead to cirrhosis in a small—but not negligible—percentage of cases (up to 8% of cases). A specific diagnostic laboratory assay for the diagnosis of hepatobiliary involvement in sarcoidosis is lacking, but an elevation of liver enzymes (especially of alkaline phosphatase (ALP) from 5–10 times the upper limit of normal) can be noted, even if its degree does not correlate with the severity of liver replacement by sarcoidosis [16].

Unfortunately, up to 80% of patients affected by liver sarcoidosis are asymptomatic [19]. Therefore, due to the lack of specific laboratory tests, the use of a unique non-invasive imaging modality—such as MRI—to assess the hepatobiliary involvement in sarcoidosis would be extremely useful to prevent the final step of liver dysfunction. To the best of our knowledge, in the scientific literature, there is a lack of reported cases presenting a complete set of MRI images, which could guide the radiologist to a confident diagnosis of hepatobiliary sarcoidosis, even in asymptomatic patients with negative liver laboratory tests.

The present study describes a case of hepatic sarcoidosis involving the biliary tract investigated via CT, magnetic resonance cholangiopancreatography (MRCP), and functional MRCP performed after infusion of gadolinium ethoxybenzyl diethylenetriamine pentaacetic acid (Gd-EOB-DTPA).

## 2. Case Presentation

**Figure 1:**
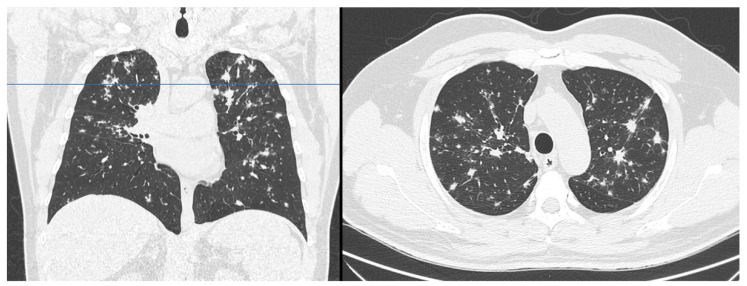
Chest computed tomography showed multiple peribronchovascular nodules, mainly located in the upper lobes.

**Figure 2:**
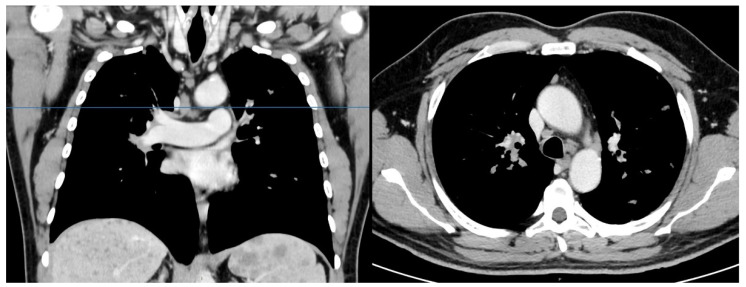
Chest computed tomography showed some slightly enlarged mediastinal lymph nodes.

**Figure 3:**
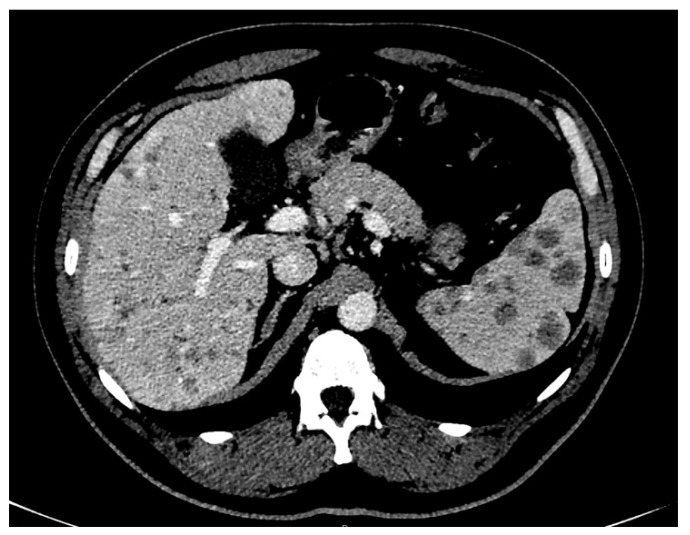
Abdominal contrast-enhanced computed tomography, in portal phase, showed multiple nodules ranging from 1.0 or 2.0 mm to 0.75 cm in diameter diffusely distributed in the liver, and ranging from 1.0 mm to 2.0 cm in the spleen.

A 53-year-old Caucasian male was admitted to our hospital on 24 April 2020, reporting a 2-month history of dyspnea triggered by moderate efforts (on aerosol therapy with no benefit) that had become associated, over the previous few days, with oppressive and persistent thoracic discomfort, both of which were exacerbated by exertion. The patient, with I-class obesity (BMI = 32), suffered from gastroesophageal reflux disease and hypertension (on medication). The electrocardiogram (ECG) showed first-degree atrioventricular (AV) block, and echocardiography demonstrated mild parietal hypertrophy compatible with the known cardiovascular anamnesis. Chest radiography showed multiple bilateral, ill-defined nodular opacities in the upper lung fields, and slight enlargement of both hilar regions. Nasopharyngeal swab excluded 2019 novel coronavirus (SARS-CoV-2) infection. A chest CT scan was performed, demonstrating peribronchovascular nodules arranged in clusters (Figure 1 and Figure 2). In the lower slices of the acquired field of view, multiple widespread hypodense nodules in the whole liver and spleen (both megalic) were identified, ranging from 1–2 mm to 2 cm (Figure 3). Therefore, the exam was extended to a total-body CT scan, which confirmed the findings mentioned above, and revealed lymphadenopathy in the chest and slightly enlarged lymph nodes in the celiac, paraaortic, and iliac stations.

**Figure 4:**
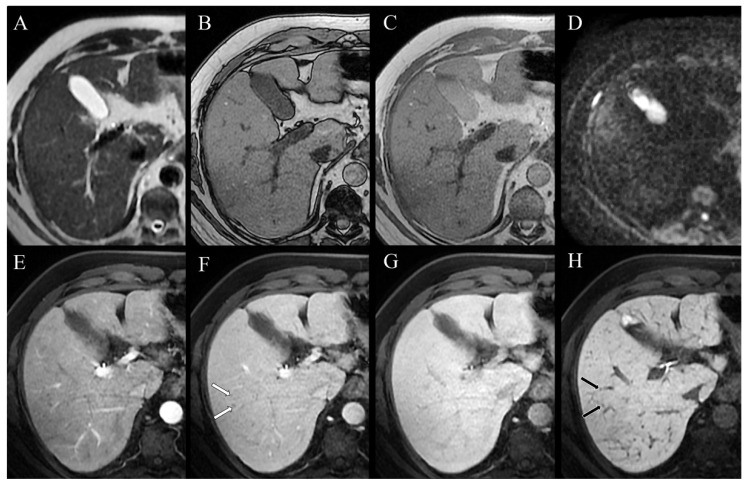
Abdomen magnetic resonance: (**A**) T2w, (**B**) T1w out-of-phase, (**C**) T1w in-phase, (**D**) DWI, (**E**) arterial phase, (**F**) portal venous phase, (**G**) delayed phase, and (**H**) hepatobiliary phase images. Millimeter-sized lesions spread throughout the whole liver appear hypointense in all sequences, and do not show enhancement after administration of contrast medium.

The differential diagnosis included tuberculosis, sarcoidosis, and lymphoma. The QuantiFERON-TB test was negative, excluding the hypothesis of tuberculosis in a patient with no history or signs of this disease. Laboratory tests demonstrated elevated levels of angiotensin-converting enzyme (135 U/L, with normal values of 13–64 U/L) and neuron-specific enolase (16.6 µg/L, with normal values < 12.5 µg/L), whereas alpha-fetoprotein (AFP), carcinoembryonic antigen (CEA), carbohydrate antigen (CA19-9), and viral hepatitis serology were negative, as well as the complete blood count. Furthermore, an elevation of ALP level was detected (240 U/L, normal values of 30–120 U/L), supporting the hypothesis of biliary involvement due to the lesions demonstrated by CT. Subsequently, the patient underwent 18F-FDG-PET/CT, which showed increased 18F-FDG uptake in all of the parenchymal lesions and lymph nodes revealed by CT, including the diffuse hepatobiliary lesions. Focal uptakes were also detected in districts where the CT scan did not reveal evident lesions, such as the laterocervical lymph nodes, heart (subsequently confirmed by cardiac MRI), lumbar spine, left femur bone marrow, and in a subdiaphragmatic area in the liver. Color Doppler sonography and transient elastography (TE, FibroScan ®, Echosens, Paris, France) revealed liver fibrosis and slow portal flow. Hence, the need to confirm the hypothesis of sarcoidosis, excluding with certainty the possibility of lymphoma, coupled with the need to confirm the nature of hepatobiliary lesions and possible indirect signs of portal hypertension required to ascertain the hepatic venous pressure gradient and perform transjugular liver biopsy. The first examination excluded portal hypertension, and the histological evaluation confirmed the hepatobiliary sarcoid involvement by demonstrating non-necrotizing granulomas. Therefore, all of the lesions highlighted by imaging were attributed to the final diagnosis of sarcoidosis. The patient was then treated with prednisone, with significant improvement in pulmonary symptoms and normalization of ALP level. Since this normalization does not necessarily correlate with regression of hepatobiliary sarcoidosis, MRI was chosen as the follow-up imaging modality of the hepatobiliary lesions. MRI, MRCP, and functional MRCP after infusion of Gd-EOB-DTPA (Primovist®, Bayer HealthCare Pharmaceuticals, Berlin, Germany) confirmed innumerable lesions in the spleen and the liver, but dimensionally and numerically reduced if compared to the previous CT scan. This feature ensured that the decrease in ALP levels was linked to the reduction in sarcoidotic hepatobiliary involvement. Sarcoidotic granulomas, widespread in the liver, showed a low signal on T1-weighted and T2-weighted images, no restriction on diffusion-weighted images (DWIs), and appeared hypointense before and after infusion of contrast medium (Figure 4A–H).

**Figure 5:**
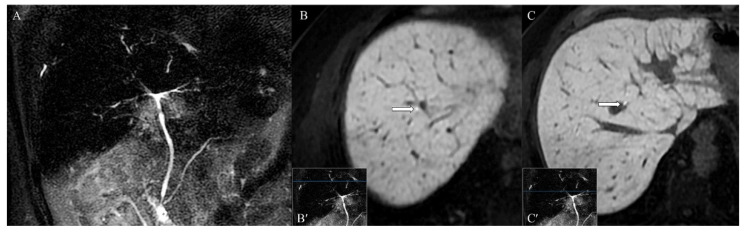
(**A**) MRI cholangiography phase, in the absence of anamnesis, does not allow a confident differential diagnosis with primary sclerosing cholangitis. (**B**,**C**) After administration of Gd-EOB-DTPA, hepatobiliary phase images (**B**) above and (**C**) below the stenosis demonstrate physiological opacification of the intrahepatic bile ducts and, despite the stenosis, physiological contrast flow.

Furthermore, the course of few intrahepatic bile ducts appeared focally restricted, without upstream dilation (Figure 5A). The functional MRCP demonstrated that the excretion of the contrast medium in the biliary tracts was maintained in the physiological time (Figure 5B,C).

**Figure 6:**
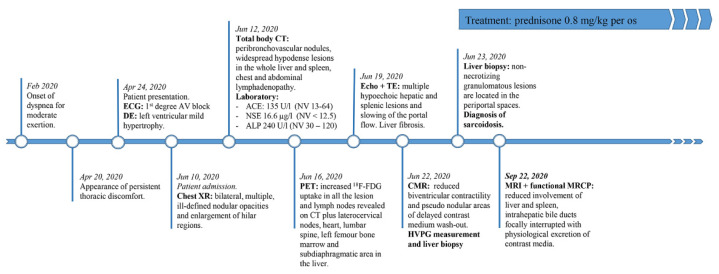
Information timeline. Abbreviations—ECG: electrocardiogram; DE: Doppler echocardiography; XR: X-ray; LYM: lymphocytes; NV: normal values; CT: computed tomography; ACE: angiotensin-converting enzyme; NSE: neuron-specific enolase; ALP: alkaline phosphatase; PET: positron emission tomography; Echo: echography; TE: transient elastography; CMR: cardiovascular magnetic resonance; HVPG: hepatic venous pressure gradient; MRCP: magnetic resonance cholangiopancreatography.

At the time of writing, treatment with immunotherapy is ongoing (Figure 6).

## 3. Discussion

This study presents a patient with multiorgan sarcoidosis and, therefore, with hepatobiliary involvement, but only complaining of dyspnea and thoracic discomfort. In patients with systemic sarcoidosis, liver dysfunction is not a rare event, and has been estimated to be present in less than one-third of those affected by chest symptoms alone [20]. Unfortunately, hepatobiliary involvement in sarcoidosis can be challenging to demonstrate, due to the non-specific clinical, imaging, and laboratory findings [21], and is confirmed only after the use of invasive techniques such as liver biopsy. Furthermore, in histopathology, granulomatous involvement of the liver is reported in approximately 40–70% of patients with systemic disease and laboratory and/or imaging signs of hepatobiliary involvement [22]. Finally, invasive techniques such as biopsy demonstrate unsatisfactory sensitivity. Therefore, it is essential to use a non-invasive imaging technique to explore liver parenchyma in these patients.

On CT scans, granulomatous nodules appear hypodense compared to the surrounding parenchyma, both before and after administration of contrast medium, showing less enhancement than the adjacent tissue [23]. In the present case, a CT scan detected the hepatobiliary involvement in sarcoidosis as multiple hypodense nodules ranging from 1–2 mm to 2 cm at the patient’s presentation. In the literature, the rate of detectability of granulomatous hepatic lesions by CT differs between different series: Warshauer reported that only 5% of hepatic sarcoidosis lesions were detectable by CT [24], whereas Fetzer demonstrated a rate of 25.6% [25]. In the Warshauer series [24], the dimensions of liver lesions were included in the resolution power of the CT. On the other hand, in the cases with negative liver findings, the size of the granulomatous lesions in the other organs was smaller—around 1.5 mm. Therefore, it is safe to assume that the hepatic parenchyma was also involved, but with even smaller lesions—not detectable by CT. Unfortunately, the authors performed only six liver biopsies, probably in CT-positive patients: the immediate consequence was that the liver’s involvement was probably higher than reported. This hypothesis seems to be supported by the findings of Fetzer. In fact, among 39 patients with liver sarcoidosis (histological diagnosis in 70% of cases), ~23.3% demonstrated CT positivity [25]. Pathologically, the physiological imaging appearance of the liver in sarcoidosis is due to the microscopic dimensions of granulomas, which become identifiable only after they coalesce into larger, macroscopic masses, ranging from 1–2 mm to several centimeters in size [24]. Therefore, in most cases, hepatic sarcoid disease cannot be detected by CT imaging. 

Furthermore, in our study, an MRI of the liver was performed as a follow-up after prednisone therapy. To the best of our knowledge, there is only one other case in the English literature [22] in which an MRI of the liver was performed as a post-therapy follow-up in hepatic sarcoidosis. The differences between our experience and that of Jung et al. are that the latter used superparamagnetic iron oxide as the contrast medium instead of Gd-EOB-DTPA, which we used in our study, and that they did not perform MRCP. In our case, liver lesions showed a low signal in both T2-weighted and T1-weighted sequences, with no signal drop between in- and out-of-phase, and no diffusion restriction on DWIs. Furthermore, after the administration of the Gd-EOB-DTPA contrast medium, no lesion enhancement was appreciable in any phase of the study. In the literature, hepatobiliary sarcoidotic lesions are reported to be slightly hypo-isointense on T1-weighted images, present a low signal on T2-weighted sequences, and not show notable enhancement after contrast medium administration [26]. However, a hyperintense T2 signal has also been reported in the event of active inflammation [22,27], size increase, and coalescence of a 6 cm granuloma [22]. Being aware of this signal pattern may be helpful in differentiating sarcoid lesions from hepatic cancer nodules or metastases, the latter tending to demonstrate higher signal intensity on T2-weighted fat-saturated sequences [28]. Moreover, in the case of diffuse hepatobiliary involvement in sarcoidosis, a reported finding of biliary involvement is the evidence of high T2 signal intensity in periportal areas [26,29], due to the tendency of granulomas to concentrate in the periportal space, thickening it [30], and leading to focal narrowing of the biliary tract. Our case is consistent with previous reports concerning radiological findings. In particular, our patient’s lesions’ low signal on T2-weighted sequences and DWIs was attributable to the good response to prednisone therapy, which reduced both inflammation activity and granuloma size.

Furthermore, in the present case, an MRCP for evaluating the biliary tree highlighted focal narrowing of intrahepatic bile ducts, without upstream dilations. To the best of our knowledge, there is only one other case in the English literature [31] reporting a diagnostic MRCP imaging finding characterized by biliary ducts narrowing due to sarcoidosis. However, the narrowing was in the extrahepatic bile duct, as opposed to our case, in which the stenosis involved intrahepatic bile ducts. Nevertheless, the study by Farooq [31]—reporting a jaundiced patient with no pulmonary lesions, and a positive CT finding of an isolated macroscopic hepatic hilar granuloma—is not comparable with the present case, as it concerned a rare presentation of this disease. In contrast, as more frequently reported in hepatobiliary sarcoidosis, our patient was asymptomatic, and showed a widespread involvement of the liver. In the literature, other cases of biliary duct stenosis due to hepatobiliary involvement in sarcoidosis are described [31,32,33,34,35,36,37,38,39,40,41,42,43,44,45]. However, all of the above were demonstrated by invasive techniques such as endoscopic retrograde cholangiopancreatography (ERCP) or percutaneous transhepatic cholangiography (PTC). All of these patients presented icterus, and all but one case demonstrated at least one stenosis detectable by ERCP due to its location in the extrahepatic biliary tree. In our study, the patient had no gastroenterological symptoms, and the intrahepatic narrowing of the biliary ducts would be hard to identify with ERCP. Moreover, MRCP was preferred to ERCP, given the patient’s clinical and laboratory conditions being in improvement. The infrequent use of MRCP in patients affected by sarcoidosis with hepatobiliary involvement—and, therefore, the lack of reported MRCP imaging findings in the literature—is probably due to the recent introduction of this examination for the evaluation of the biliary tree, along with the absence of comparative studies investigating the diagnostic accuracy of MRCP versus ERCP and PTC in sarcoidosis. 

Moreover, our case is the first in the literature to present a functional MRCP evaluation of the biliary ducts in a patient with diagnosed systemic sarcoidosis and hepatobiliary involvement after endovenous administration of Gd-EOB-DTPA. The use of this contrast medium in the study of the biliary tree is off-label, but it is very helpful in the differential diagnosis of focal liver lesions [46]. Therefore, in the case of the study of unknown hepatobiliary lesions, the use of this new contrast medium allows us to obtain high diagnostic performances on both the hepatic parenchyma and the biliary tract [46]. Furthermore, in patients diagnosed with sarcoidosis—such as ours—according to a recent meta-analysis, the relative risk of developing liver cancer is not negligible [47]. Furthermore, the imaging of the bile ducts obtained in the delayed phase of the study allowed us to rule out primary sclerosing cholangitis based on the focal distribution of the narrowings, the absence of upstream dilations, and the evidence of contrast material proximal and distal to the apparent filling defects, indicating that there were no flow-limiting lesions.

In the present case, the patient’s liver was studied using in- and out-of-phase T1-weighted sequences, T2-weighted sequences, and DWIs. Furthermore, after intravenous injection of the contrast medium, images of arterial and portal venous phases, 20 minutes delayed, as well as hepatobiliary phases were acquired. Five other cases in the literature report MRI findings of patients diagnosed with sarcoidosis and hepatobiliary involvement [22,27,48,49,50]; however, unlike the present case, none of these studies reports all of the sequencing images used for the characterization of the lesions. All of the authors of these papers reported only a partial set of images; the reason for this is that they probably only focused on specific aspects of the disease in their patients’ livers. 

A possible limitation of our case was the absence of a baseline MRI before therapy. However, our patient did not undergo magnetic resonance at presentation because the CT scan that first identified the hepatobiliary lesions was performed in the emergency room of our institution, and there were no criteria for the urgent execution of an MRI at that moment. Furthermore, following the histological findings, no further imaging investigations were required before the beginning of the therapy. Finally, the post-therapy follow-up was performed directly by MRI due to its non-invasiveness and safety. Since sarcoidosis can affect young people, using a diagnostic tool that does not expose the patient to ionizing radiation should be mandatory.

## 4. Conclusions

In conclusion, imaging plays a crucial role in evaluating the liver parenchyma and biliary tree in patients affected by sarcoidosis, allowing for early assessment of hepatobiliary involvement that might significantly change patient outcomes. This study provides a complete set of MRI, MRCP, and functional-MRCP images after infusion of Gb-EOB-DTPA, representing a valuable guide for radiologists to reach a confident diagnosis of hepatobiliary involvement in sarcoidosis.
